# Can patient and family education prevent medical errors? A descriptive study

**DOI:** 10.1186/s12913-020-05083-y

**Published:** 2020-03-31

**Authors:** Yoon-Sook Kim, Hyuo Sun Kim, Hyun Ah. Kim, Jahae Chun, Mi Jeong Kwak, Moon-Sook Kim, Jee-In Hwang, Hyeran Kim

**Affiliations:** 1 Department of Quality Improvement, Konkuk University Medical Centre, 120-1 Neungdong-ro (Hwayang-dong), Gwangjin-gu, Seoul, 05030 South Korea; 2 Performance Improvement Team, Uijeongbu St. Mary’s Hospital, Uijeongbu, South Korea; 3 Office of Quality Innovation, Samsung Medical Center, Seoul, South Korea; 4 Office of QI, Severance Hospital, Seoul, South Korea; 5 Quality Improvement Team, Korea University Anam Hospital, Seoul, South Korea; 6 Medical Nursing Department, Seoul National University Hospital, Seoul, South Korea; 7 Kyung Hee University College of Nursing Science, Seoul, South Korea

**Keywords:** Patient and family education, Educational content, Medical error, Patient safety officer

## Abstract

**Background:**

This study aims to increase understanding of how patient and family education affects the prevention of medical errors, thereby providing basic data for developing educational contents.

**Methods:**

This descriptive study surveyed patients, families, and Patient Safety Officers to investigate the relationship between educational contents and medical error prevention. The Chi-square test and ANOVA were used to derive the results of this study. The educational contents used in this study consisted of health information (1. current medicines, 2. allergies, 3. health history, 4. previous treatments/tests and complications associated with them) and Speak Up (1. handwashing, 2. patient identification, 3. asking about medical conditions, 4. asking about test results, 5. asking about behaviour and changes in lifestyle, 6. asking about the care plan, 7. asking about medicines, and 8. asking about medicine interactions).

**Results:**

In this study, the first criterion for choosing a hospital for treatment in Korea was ‘Hospital with a famous doctor’ (58.6% patient; 57.7% families). Of the patients and their families surveyed, 82.2% responded that hospitals in Korea were safe. The most common education in hospitals is ‘Describe your medical condition’, given to 69.0% of patients, and ‘Hospitalisation orientation’, given to 63.4% of families. The most important factors in preventing patient safety events were statistically significant differences among patients, family members, and Patient Safety Officers (*p* = 0.001). Patients and families had the highest ‘Patient and family participation’ (31.0% of patients; 39.4% of families) and Patient Safety Officers had the highest ‘Patient safety culture’ (47.8%).

**Conclusions:**

Participants thought that educational contents developed through this study could prevent medical errors. The results of this study are expected to provide basic data for national patient safety campaigns and standardised educational content development to prevent medical errors.

## Background

### What is patient safety?

Patient safety has become an important issue following the reporting of the status and improvement plan of patient safety in the ‘To err is human’ report published by the Institute of Medicine (IOM) in 1999 [1]. The IOM defines terms related to patient safety as follows [1]: ‘medical error’ is defined as not performing the planned care as intended or performing the wrong care plan.; ‘adverse event’ refers to an injury occurring during a medical treatment; and ‘near-miss’ is defined as the occurrence of a medical error but no event impacting the patient and no harmful consequences. The IOM defined patient safety as a patient being free from incidental injury. The World Health Organization defines it as ‘the absence of preventable harm to a patient and reduction of risk of unnecessary harm associated with health care to an acceptable minimum’ [2]. In other words, patient safety means preventing the harm that may occur through incidental or preventable injury from medical treatment, including overlooking appropriate treatment [3].

### Why is patient safety important?

Medical errors can cause serious harm to patients and negatively impact medical institutions both qualitatively (e.g. patient safety and quality of care, hospital’s social image) and quantitatively (e.g. medical dispute costs, patient compensation costs, number of patients, and medical income) [4]. Many medical errors occur infrequently, but they can shorten life or accelerate impending death. Medical errors are so frequent that they are estimated to be the third most common cause of death in the United States [5]. As such, medical errors are a major concern of national and medical institutions because they are directly related to the patients’ life.

### How to improve patient safety

Patients and families are considered part of the ‘care team’ and are encouraged to engage in treatment and safety [6]. Patient involvement improves patient safety by reducing medical errors [7, 8]. If patients participate in the medical process, they have improved health literacy, self-care, and compliance with doctor’s instructions (in particular, medication compliance) [9, 10]. This improves the patients’ treatment results and satisfaction [11].

### How to involve patients and family

Patient participation is defined as ‘the degree of behavioural, emotional, and informational effort to provide patients with the information for healthcare need in the care process’ [12]. In addition, patient participation means that the patient or family is involved in preventing or reporting a medical error related to the care [13, 14]. Patient participation methods are as follows: asking health care providers about their medical condition, gathering medical information, implementing recommended medical instructions, and exploring and making decisions on alternative treatments [15].

Patient participation-related studies have reported specific error-based (e.g. hospital-acquired pressure ulcers [16], falls [17, 18], and medicine [19]), disease-based [20–22], and patient-safety culture-based (e.g. Communication [23], Speak Up [24], and patient safety campaign [25]). Patient Safety Officers provide patients and families with personal patient education, large patient-safety campaigns, brochures, patient-safety videos, and other resources to increase patient engagement. As such, Patient Safety Officers are educated in various ways, but there are not many studies evaluating their effectiveness.

The purpose of this study was to present relevant evidence through comparative studies between educational content and the degree of prevention of medical errors. Thus, we want to provide Patient Safety Officers with basic data in developing educational contents to improve patient engagement.

## Methods

### Research design

This was a descriptive study examining the relationship between educational content and the degree to which medical errors can be prevented.

### Participants and data collection

A total of 242 participants included 58 patients, 71 families, and 113 Patient Safety Officers.

The patients or family who participated in the study were those who visited two tertiary hospitals in Seoul and one general hospital in Gyeonggi-do. Patients’ and families’ data were collected through a survey from October 10, 2018, to November 10, 2018. Data collection was conducted after the trained investigator explained the purpose of the survey face-to-face to the patient or family and received informed consent to participate in the study.

The Patient Safety Officers involved in the study work on patient safety and quality improvement at the hospitals. Patient Safety Officers are those who have agreed to participate in research among those attending the Korean Quality Improvement Nurses Society Conference (October 25, 2018). We asked Patient Safety Officers to explain the purpose and method of the study and to fill out a self-report questionnaire. We also received informed consent from Patient Safety Officers to participate in the study.

### Measures

The questionnaire used in this study was developed through the following process.

- 1st step: Investigate patient safety-related organisations (Healthcare Research and Quality, Institute for Healthcare Improvement, Canadian Patient Safety Institute, World Health Organization) [26]
- 2nd step:
  1. Seven internal researchers extracted 10 items related to patient and family participation from the data investigated in step 1: Current medicines, allergies, health history, hand-washing, and asking about medical condition, behavioural and lifestyle change, care plan, medicines, medical interaction, discharge and/or transitional care plan.
  2. Internal researchers consisted of a nursing professor, nursing director, and Patient Safety Officers with at least five years of experience in patient safety and quality improvement.
- 3rd step
  1. The items extracted in step 2 were translated into Korean.
  2. The translated questionnaire was finalised with the advice of an expert with over 10 years of experience in patient safety and quality improvement.
  3. Experts recommended dividing the category into two (providing health information, speak up), deleting one duplicate item (asking about the discharge and/or transitional care plan), and adding three items (previous treatments/tests and complications associated with them, patient identification, asking about test results).

The questionnaire consisted of patient and family characteristics, Patient Safety Officer characteristics, most necessary elements to prevent medical errors, and educational contents (providing health information and speak up). The reliability of the study questionnaire was .973 for educational contents (.916 for providing health information and .839 for speak up). The variables are described in Additional file 1: Appendix A (questionnaire; See Additional file 1: Appendices B and C).

### Data analysis

The collected data were analysed using IBM SPSS Statistics 24, setting *p* < .05 as the cut-off for statistical significance.

Statistical analysis is as follows:

- Cronbach’s α: reliability analysis of questionnaire questions regarding educational contents (to ensure that the same results can be obtained for repeated measurements)
- Mean and standard deviation: age, Patient Safety Officer’s career duration;
- Chi-square test
  1. General characteristic differences between patients and their families
  2. Difference according to educational experience of Patient Safety Officers
  3. Differences in the most necessary element to prevent medical errors between the groups
- ANOVA
  1. Differences in the most necessary element to prevent medical errors between the three groups
  2. Difference in the degree of medical error prevention between the three groups

## Results

### Are there differences in general characteristics between patients and families?

The mean age of patients was 51.53 (SD 14.71), OPE was 91.4%, and IPE was 72.4%. The mean age of families was 46.58 (SD 12.80), OPE was 71.8%, and IPE was 29.6%. There was a statistically significant difference between the two groups in OPE (*p* = 0.007) and IPE (*p* < 0.001; see Table 1).

**Table 1:** General characteristic differences between the patients and family

	Total, *n* = 129		Patient, *n* = 58		Family, *n* = 71		*P*
	n	%	n	%	n	%	
Age, years							
≤ 39	45	34.9	16	27.6	29	40.8	0.079
40–49	29	22.5	11	19.0	18	25.4	
≥ 50	55	42.6	31	53.4	24	33.8	
OPE (y)	104	80.6	53	91.4	51	71.8	0.007^**^
IPE (y)	63	48.8	42	72.4	21	29.6	0.000^***^
Criteria for selecting hospital							
Large hospital	24	18.6	13	22.4	11	15.5	0.631
Hospital with a famous doctor	75	58.1	34	58.6	41	57.7	
Hospital near home	10	7.8	4	6.9	6	8.5	
Hospital centred on patient safety	20	15.5	7	12.1	13	18.3	
Is the hospital safe? (y)	106	82.2	47	81.0	59	83.1	0.819
Education received at the hospital							
How to identify patients (y)	81	62.8	39	67.2	42	59.2	0.366
Handwashing methods (y)	73	56.6	35	60.3	38	53.5	0.478
How to prevent falls (y)	82	63.6	38	65.5	44	62.0	0.718
Hospitalisation orientation (y)	83	64.3	38	65.5	45	63.4	0.855
Take all your medicines to your doctor visit (y)	72	55.8	31	53.4	41	57.7	0.722
Describe your medical condition (y)	82	63.6	40	69.0	42	59.2	0.274
Participate in your care plan (y)	76	58.9	36	62.1	40	56.3	0.560
Bring a list of questions to your doctor visits (y)	37	28.7	12	20.7	25	35.2	0.081

^**^p < 0.01, ^***^p < 0.001; *y* yes, *OPE* outpatient experience, *IPE* inpatient experience

When asked what to consider first when choosing a hospital for treatment, the majority of both patients (58.6%) and families (57.7%) answered ‘Hospital with a famous doctor’. On the other hand, 82.2% of patients and families thought the hospital was safe. The highest response rates for education received in hospitals were ‘Describe your medical condition’ for 69.0% of patients and ‘Hospitalisation orientation’ for 63.4% of families, while the lowest response rates were both ‘Hospitalisation orientation’ (20.7% of patients, 35.2% of families).

### Is there a difference in the characteristics of the patient safety officer according to educational experience?

The mean age of Patient Safety Officer was 41.83 (SD 8.21). Their mean career duration was 4.78 with PFE (SD 4.79) and 2.12 without PFE (SD 2.29). There was a statistically significant difference between the two groups (*p* = 0.001; see Table 2). On the most effective educational methods were ‘Speak up’, (47.8%), followed by ‘face-to-face’ (37.2%).

**Table 2:** Difference according to educational experience of Patient Safety Officer

	Total, *n* = 113		PFE experience				*P*
			With, *n* = 58		Without, *n* = 55		
	n	%	n	%	n	%	
Age, years							
≤ 39	43	38.1	20	34.5	23	41.8	0.319
40–49	47	41.6	23	39.7	24	43.6	
≥ 50	23	20.4	15	25.9	8	14.5	
Career duration of Patient Safety Officer, years							
< 1	19	22.1	4	8.2	15	40.5	0.001^**^
1–3	18	20.9	11	22.4	7	18.9	
≥ 3	49	57.0	34	69.4	15	40.5	
The most effective method of PFE							
Speak up	54	47.8	28	48.3	26	47.3	0.345
Face-to-face	42	37.2	23	39.7	19	34.5	
Campaign	6	5.3	4	6.9	2	3.6	
Media	11	9.7	3	5.2	8	14.5	

^**^p < 0.01; *PFE* Patient and Family Education

### The most necessary elements to prevent medical errors

The most important factors in preventing patient safety events were statistically significant differences among patients, family, and Patient Safety Officer (*p* = 0.001; see Table 3). Patients and families had the highest ‘Patient and family engagement’ (31.0% of patients; 39.4% of families) and Patient Safety Officer had the highest ‘Patient safety culture’ (47.8%).

**Table 3:** Differences in the most necessary element to prevent medical errors between the three groups

	Total, *n* = 242		Patient, *n* = 58		Family, *n* = 71		Patient Safety Officer, *n* = 113		*P*
	n	%	n	%	n	%	n	%	
Patient and family engagement	72	29.8	18	31.0	28	39.4	28	23.0	0.001^**^
Employees’ patient safety activities	36	14.9	9	15.5	18	25.4	9	8.0	
Patient safety culture	90	37.2	19	32.8	17	23.9	54	47.8	
Budget support	44	18.2	12	20.7	8	11.3	24	21.2	

**p < 0.01

### Patient and family education prevents medical errors

As shown in Table 4, there was no statistically significant difference between the three groups in the idea that educational content prevents medical errors. This was not statistically different, because all groups responded with high scores indicating that educational content can prevent medical errors. The average score was over 4.0 in all items, and the information on ‘allergies’ was the highest, with an average of 4.51.

**Table 4:** Difference in the degree of medical error prevention between the three groups

	Total, *n* = 242	Patient, *n* = 58	Family, *n* = 71	Patient Safety Officer, *n* = 113	*p*
	Mean ± SD	Mean ± SD	Mean ± SD	Mean ± SD	
**Providing health information**					
Current medicines	4.31 ± 0.96	4.29 ± 1.23	4.32 ± 1.01	4.32 ± 0.77	0.982
Allergies	4.51 ± 0.88	4.50 ± 1.06	4.45 ± 0.94	4.55 ± 0.74	0.764
Health history	4.42 ± 0.90	4.62 ± 0.93	4.41 ± 0.98	4.33 ± 0.81	0.126
Previous treatments/tests and complications associated with them	4.44 ± 0.90	4.60 ± 0.95	4.38 ± 1.02	4.39 ± 0.77	0.273
**Speak Up**					
Handwashing	4.27 ± 1.04	4.16 ± 1.21	4.32 ± 1.12	4.30 ± 0.89	0.609
Patient identification	4.47 ± 0.97	4.29 ± 1.20	4.37 ± 1.09	4.62 ± 0.72	0.067
Asking about medical condition	4.27 ± 0.93	4.38 ± 1.17	4.32 ± 0.92	4.18 ± 0.78	0.338
Asking about test results	4.40 ± 0.86	4.48 ± 1.00	4.37 ± 0.98	4.39 ± 0.70	0.722
Asking about behaviour and change in lifestyle	4.40 ± 0.85	4.52 ± 1.00	4.49 ± 0.84	4.28 ± 0.76	0.130
Asking about the care plan	4.39 ± 0.88	4.50 ± 1.03	4.41 ± 0.94	4.32 ± 0.75	0.431
Asking about medicines	4.47 ± 0.83	4.55 ± 0.96	4.54 ± 0.84	4.39 ± 0.74	0.354
Asking about medicine interactions	4.35 ± 0.92	4.45 ± 1.11	4.37 ± 0.98	4.28 ± 0.77	0.531

*SD* standard deviation

## Discussion

We believed that patient safety can be more thoroughly addressed by encouraging patient safety knowledge and the understanding of unique concerns associated with their health, such as patient and family engagement in safety [27]. Patient safety programmes emphasise patient and family engagement and actively prevent safety issues by focussing on individual respect, patient rights, and care coordination [13, 14]. A patient safety campaign is useful for optimising situations in which interventions require medical staff-patient communications on safety [28].

As mentioned earlier, there have been many studies on medical errors; however, few studies have addressed education targeting patient, family, and Patient Safety Officer. Patient and family engagement in patient safety activities is very important. Thus, a Patient Safety Officer should develop educational contents to increase patient and family engagement in patient safety activities.

In this study, we drew upon educational material from the Healthcare Research and Quality, Institute for Healthcare Improvement, and Canadian Patient Safety Institute to develop a measure for preventing medical errors. The priority for medical error prevention for patients and families was ‘patient and family engagement’, whereas Patient Safety Officer emphasised establishing a ‘Patient safety culture’ within the hospital. Patients and their families perceive that their role is important in preventing medical errors, while Patient Safety Officer recognise that when a culture is formed within an organisation, it can lead to the active participation of all staff, including executives.

Korea’s health institution accreditation’s standards require that patient safety education be provided to patients and their family. According to the accreditation’s standards, inpatients receive education through face-to-face, videos, and so on, and confirm that they understand the education. However, outpatients may only receive information through post or a leaflet.

In Korea, most of the education provided by the hospital is received by the family and delivered to the patient. Nevertheless, the recent enactment of the Patient Safety Act has increased the public and Patient Safety Officers’ interest in patient safety. Therefore, patients, family members, and Patient Safety Officers thought that any education related to patient safety would be effective, and the results of this study showed high scores.

The formation of a patient safety culture in hospitals is recognised as ‘key to quality improvement’, [29] and one study shows the importance of this culture in efforts to reduce medical error [30]. Patients and families who participated in our study emphasised being informed of changes to prescribed medicines and ‘Providing health information’ on what medicine the patient is currently taking, indicating that medical errors could be prevented if patient and their family ensure ‘Providing health information’ accurately. On the other hand, Patient Safety Officer emphasised the importance of asking medical staff whether they have confirmed the patient’s identity. In addition, patient and family indicated that accurately ‘Providing health information’ on patient allergies to medical staff was important for preventing medical errors. The results of this study indicate that the patient and family focus on influencing the patient’s health condition, while Patient Safety Officers focus on assuring patient safety by using health information of patients that the medical staff has not confirmed.

Korea did not recognise the importance of PFE or patient safety activities in hospital until the implementation of the Patient Safety Act in 2016. Under the Act, the Patient Safety Officer should conduct PFE through patient safety activities, but they did not have knowledge about the education for patient engagement. As a result, Patient Safety Officers provided patients with healthcare provider-centred education rather than patient-centred education. Thus, the same medical errors occurred repeatedly, and patient safety incident reports did not decrease.

Nearly all participants in this study provided a score of no less than 4 for the ‘Providing health information’ and ‘Speak Up’ variables; thus, they clearly recognised the urgent need in Korea to prevent medical errors.

In Korea, it is necessary to develop and present standardised educational contents at the national level, which should be informed by this study’s results. Korea should actively promote the importance of patient and family engagement, Patient Safety Officer, hospitals, and citizens for preventing medical errors.

## Conclusions

Under Article 5 of Korea’s Patient Safety Act, ‘patients and their protectors shall participate in patient safety activities’. In addition, Article 12 requires that Patient Safety Officer should perform ‘education of patients and their protectors for their patient safety activities’. This indicates that PFE is the basis of patient safety activities in Korea.

In the Organisation for Economic Cooperation and Development’s 2012 Health Care Quality Review [31], the Republic of Korea was reported to have no clear mechanism to ensure patient safety. Since then, the National Patient Safety Committee has been established, together with a national patient safety reporting system; however, no standardised PSE contents for medical error prevention have been provided to date. Moreover, the patient safety reporting system established to prevent and educate on medical errors is not properly utilised.

The study suggests that patient safety education involving patients, families, and Patient Safety Officers can all prevent medical errors. A method should be developed to ensure patient safety through patients rather than unilateral delivery to them. It is hoped that the educational contents developed based on data from the AHRQ, IHI, and CPSI will spread awareness regarding patient safety at national level, and promote the development of standardised educational contents.

## Supplementary information

**Additional file 1: Appendix A.** Variables description. **Appendix B.** Informed consent for study on Patient Safety Education Needs (For patient and family). **Appendix C.** Informed consent for study on Patient Safety Education Needs (For Patient Safety Officer).

## Data Availability

The dataset used and analysed during the current study is available from the corresponding author on reasonable request.

## Abbreviations

IOM: Institute of Medicine
IPE: Inpatient experience
OPE: Outpatient experience
PFE: Patient and family education
