# Supplementary material for: Can patient and family education prevent medical errors? A descriptive study
Source: BMC Health Serv Res. 2020 Mar 31;20:269. doi: 10.1186/s12913-020-05083-y (PMC7106564; doi:10.1186/s12913-020-05083-y)
Supplement: Supplementary file 1 — Additional file 1: Appendix A. Variables description. Appendix B. Informed consent for study on Patient Safety Education Needs (For patient and family). Appendix C. Informed consent for study on Patient Safety Education Needs (For Patient Safety Officer). [file 12913_2020_5083_MOESM1_ESM.docx]

Additional file 1: Appendix A. Variables description

| Variables | Description |
| --- | --- |
| OPE | Have you visited outpatient department for healthcare service? ‘*yes*’ or ‘*no*’  If the response is ‘*yes’*, how many times have you visited? |
| IPE | Have you ever been an admitted inpatient? ‘*yes*’ or ‘*no*’  If the response is ‘*yes’*, how many times have you been admitted? |
| Criteria to choose a hospital | If you need treatment, which hospital do you choose?  Criteria to choose a hospital was divided into four categories: (1) large hospital (tertiary hospital, university hospital, etc.), (2) hospital with a famous doctor, (3) hospital near home, (4) hospital centred on patient safe (or accredited hospital, or patient-centred hospital). |
| Is the hospital safe? | Do you think the hospital is safe enough to receive healthcare service? ‘*yes*’ or ‘*no*’ |
| Education received at the hospital | Have you ever been educated by medical staff about hospitalisation or illness? ‘*yes*’ or ‘*no*’  If the response to ‘have you ever been educated’ is ‘*yes’*, what was the educational content?  Educational content: (1) how to identify patients; (2) handwashing methods; (3) how to prevent falls; (4) hospitalisation orientation (hospital room etiquette, meal time, visit time, hospital facilities, fire prevention and evacuation tips, no smoking in hospital, etc.); (5) take all your medicines to your doctor visit (prescription medicines, vitamins, dietary or herbal supplements, etc.); (6) describe your medical condition (past history, allergy, surgery, etc.); (7) participate in your care plan; 8) bring a list of questions to your doctor visits. |
| Career duration of Patient Safety Officers | Have you done any Patient Safety Officer work? ‘*yes*’ or ‘*no*’  If the response is ‘*yes’*, how long have you worked on it?  If the response is *‘no’*, the survey was ended. |
| PFE experience | Have you provided patient and family education? ‘*yes*’ or ‘*no*’  If the response to have provided education is ‘*yes’*, what do you think is the most effective educational method? Educational methods were divided into four categories: (1) Speak up, (2) face-to-face, (3) campaign, and (4) media (video, YouTube, templates, brochures, poster, App, etc.). |
| Most necessary elements to prevent medical errors | What is most needed to prevent medical errors?  Necessary elements were divided into four categories: (1) patient and family engagement, (2) employees’ patient safety activities, (3) patient safety culture, and (4) budget support (budgeting of the hospital, National Health Insurance Fee for patient safety, National Patient Safety Campaign, etc.). |
| Type of Educational content |  |
| Providing health information | How do you think the information you provide to medical staff can prevent a medical error? Responses were rated on a 5-point scale: 1 (never) to 5 (always).  Health information: (1) current medicines (including vitamins and dietary or herbal supplements), (2) allergies (drugs, food, pollen, dust, mites, etc.), (3) health history (medical history, past surgeries, etc.), (4) previous treatments/tests and complications associated with them. |
| Speak up | How do you think your questions of medical staff can prevent a medical error? Responses were rated on a 5-point scale: 1 (never) to 5 (always).  Speak up: (1) handwashing (asking if the staff washed their hands before performing a clean or aseptic procedure), (2) patient identification (asking if patient identification was confirmed before medical practice), (3) asking about medical condition (‘What is my health problem?’ or ‘What is my diagnosis?’), (4) asking about test results (‘What is the test result?’ or ‘What are the possible complications?’), (5) asking about behaviour and changes in lifestyle (‘What should I eat?’ or ‘What exercises are good for me?’ or ‘What activities or foods should I avoid?’), (6) asking about the care plan (‘How many times will the test be performed?’ or ‘Why do I need this procedure?’ or ‘What is next my care plan step?’), (7) asking about medicines (‘What will the medicine you are prescribing do?’ or ‘Are there reactions to this medicine?’ or ‘Can you change a medicine?’ or ‘How do I take it?’ or ‘Are there any side effects?’), (8) asking about medicine interaction (‘Will this medicine interact with medicines I’m already taking?’). |

OPE, Outpatient experience; IPE, inpatient experience; PFE, Patient and Family Education.

Appendix B. Informed consent for study on Patient Safety Education Needs (For patient and family)

[Name of Principal Investigator] Yoon-Sook Kim

[Name of Organisation] Konkuk University Medical Centre

[Name of Sponsor] Korea Health Industry Development Institute

Thank you for participating in the ‘Study on Patient Safety Education Needs’

This research is being conducted by the Ministry of Health and Welfare and the Korea Health Industry Development Institute (Research Title: Development of practical reflux method for improving patient safety level).

The purpose of this study is to identify and analyse the current state of patient safety education, educational needs, and patient safety.

The survey will take about 20 minutes and there are no risks, losses, or benefits of medical conditions for those who are expected to participate in the survey. Also, under the guidance of the Research Ethics Committee, we do not collect your personal information (name, contact information, address). The survey will only be used for research purposes to develop patient and family educational content and will not be used for any other purpose.

You do not have to answer any questions that are uncomfortable, and you can stop participating in the research at any time during the investigation and there will be no disadvantages or discrimination.

-----------------------------------------------------

I have been fully informed of the survey. I have had the opportunity to ask questions about it and any questions that I have asked have been answered to my satisfaction. I consent voluntarily to participate as a participant in this research.

Print Name of Participant

Signature of Participant

Date

Day/month/year

**A. General Information**

□ Patient □ Family

A1. Age:

A2. Gender ① Female ② Male

A3. Have you visited an outpatient department for healthcare service?

① Yes (Number of times: ) ② No

A4. Have you ever been an admitted inpatient? ① Yes (Number of times: ) ② No

A5. If you need treatment, which hospital do you choose?

① Large hospital (tertiary hospital, university hospital, etc.) ② Hospital with a famous doctor ③ Hospital near home ④ Hospital centred on patient safety ⑤ Accredited hospital ⑥ Patient-centred hospital

A6. Do you think the hospital is safe enough for receiving healthcare services? ① Yes ② No

**B. State of patient safety education**

B1. Have you ever been educated by medical staff about hospitalisation or illness? ① Yes ② No

| **Educational contents** | **Educational method** | **Difficulty of understanding** | | | | | | | | |
| --- | --- | --- | --- | --- | --- | --- | --- | --- | --- | --- |
| 1) How to identify patients  □ Never been educated | ① Speak up ② Face-to-face ③ Campaign  ④ Templates, brochures, poster, etc.  ⑤ Video, YouTube, App, etc. ⑥ Other ( ) | **1** | **2** | | **3** | | | **4** | | **5** |
|  |  | 1 (Very difficult) -> 5 (Very easy) | | | | | | | | |
| 2) Handwashing methods  □ Never been educated | ① Speak up ② Face-to-face ③ Campaign  ④ Templates, brochures, poster, etc.  ⑤ Video, YouTube, App, etc. ⑥ Other ( ) | **1** | | **2** | | **3** | **4** | | **5** | |
|  |  | 1 (Very difficult) -> 5 (Very easy) | | | | | | | | |
| 3) How to prevent falls  □ Never been educated | ① Speak up ② Face-to-face ③ Campaign  ④ Templates, brochures, poster, etc.  ⑤ Video, YouTube, App, etc. ⑥ Other ( ) | **1** | | **2** | | **3** | **4** | | **5** | |
|  |  | 1 (Very difficult) -> 5 (Very easy) | | | | | | | | |
| 4) Hospitalisation orientation (hospital room etiquette, meal time, visit time, hospital facilities, fire prevention and evacuation tips, no smoking in hospital etc.)  □ Never been educated | ① Speak up ② Face-to-face ③ Campaign  ④ Templates, brochures, poster, etc.  ⑤ Video, YouTube, App, etc. ⑥ Other ( ) | **1** | | **2** | | **3** | **4** | | **5** | |
|  |  | 1 (Very difficult) -> 5 (Very easy) | | | | | | | | |
| 5) Take all your medicines to your doctor visit (prescription medicines, vitamins, dietary or herbal supplements, etc.)  □ Never been educated | ① Speak up ② Face-to-face ③ Campaign  ④ Templates, brochures, poster, etc.  ⑤ Video, YouTube, App, etc. ⑥ Other ( ) | **1** | | **2** | | **3** | **4** | | **5** | |
|  |  | 1 (Very difficult) -> 5 (Very easy) | | | | | | | | |
| 6) Tell your medical condition (past history, allergy, surgery, etc.)  □ Never been educated | ① Speak up ② Face-to-face ③ Campaign  ④ Templates, brochures, poster, etc.  ⑤ Video, YouTube, App, etc. ⑥ Other ( ) | **1** | | **2** | | **3** | **4** | | **5** | |
|  |  | 1 (Very difficult) -> 5 (Very easy) | | | | | | | | |
| 7) Participate in your care plan  □ Never been educated | ① Speak up ② Face-to-face ③ Campaign  ④ Templates, brochures, poster, etc.  ⑤ Video, YouTube, App, etc. ⑥ Other ( ) | **1** | | **2** | | **3** | **4** | | **5** | |
|  |  | 1 (Very difficult) -> 5 (Very easy) | | | | | | | | |
| 8) Bring a list of questions to your doctor visits  □ Never been educated | ① Speak up ② Face-to-face ③ Campaign  ④ Templates, brochures, poster, etc.  ⑤ Video, YouTube, App, etc. ⑥ Other ( ) | **1** | | **2** | | **3** | **4** | | **5** | |
|  |  | 1 (Very difficult) -> 5 (Very easy) | | | | | | | | |

B2. How much can you prevent medical errors when you provide the following information?

| **Items** | **medical error prevention** | | | | | **Items** | **medical error prevention** | | | | |
| --- | --- | --- | --- | --- | --- | --- | --- | --- | --- | --- | --- |
| B2-1) Current medicines (including vitamins or dietary or herbal supplements) | **1** | **2** | **3** | **4** | **5** | B2-2) Allergies (drugs, food, pollen, dust, mites, etc.) | **1** | **2** | **3** | **4** | **5** |
|  | 1 (Never) -> 5 (Always) | | | | |  | 1 (Never) -> 5 (Always) | | | | |
| B2-3) Health history (medical history, past surgeries, etc.) | **1** | **2** | **3** | **4** | **5** | B2-4) Previous treatments/tests and complications associated with them | **1** | **2** | **3** | **4** | **5** |
|  | 1 (Never) -> 5 (Always) | | | | |  | 1 (Never) -> 5 (Always) | | | | |

B3. How much can you prevent medical errors when you ask medical staffs the following items?

| **Items** | **medical error prevention** | | | | | **Items** | **medical error prevention** | | | | |
| --- | --- | --- | --- | --- | --- | --- | --- | --- | --- | --- | --- |
| C5-1) Handwashing (ask if the staff washed their hands before performing a clean or aseptic procedure) | **1** | **2** | **3** | **4** | **5** | C5-2) Patient identification (Ask if patient identification was confirmed before medical practice) | **1** | **2** | **3** | **4** | **5** |
|  | 1 (Never) -> 5 (Always) | | | | |  | 1 (Never) -> 5 (Always) | | | | |
| B C5-3) Asking about medical conditions (‘What is my health problem?’ or ‘What is my diagnosis?’) | **1** | **2** | **3** | **4** | **5** | C5-4) Asking about test results (‘What is the test result?’ or ‘What are the possible complications?’) | **1** | **2** | **3** | **4** | **5** |
|  | 1 (Never) -> 5 (Always) | | | | |  | 1 (Never) -> 5 (Always) | | | | |
| C5-5) Asking about behaviour and changes in lifestyle (‘What should I eat?’ or ‘What exercises are good for me?’ or ‘What activities or foods should I avoid?’) | **1** | **2** | **3** | **4** | **5** | C5-6) Asking about the care plan (‘How many times is the test performed?’ or ‘Why do I need this procedure?’ or ‘What is next my care plan?’) | **1** | **2** | **3** | **4** | **5** |
|  | 1 (Never) -> 5 (Always) | | | | |  | 1 (Never) -> 5 (Always) | | | | |
| C5-7) Asking about medicines (‘What will the medicine you are prescribing do?’ or ‘Are there reactions to this medicine?’ or ‘Can you change a medicine?’ or ‘How do I take it?’ or ‘Are there any side effects?’) | **1** | **2** | **3** | **4** | **5** | C5-8) Asking about medicine interaction (‘Will this medicine interact with medicines I’m already taking?’) | **1** | **2** | **3** | **4** | **5** |
|  | 1 (Never) -> 5 (Always) | | | | |  | 1 (Never) -> 5 (Always) | | | | |

B4. What is most needed to prevent medical errors?

① Patient and family engagement ② Employees’ patient safety activities ③ Patient safety culture ④ Budgeting of the hospital ⑤ Government support (National Health Insurance Fee for patient safety, National Patient Safety Campaign, etc.) ⑥ Other ( )

**Thank you for responding to our survey.**

Appendix C. Informed consent for study on Patient Safety Education Needs (For Patient Safety Officer)

[Name of Principal Investigator] Yoon-Sook Kim

[Name of Organisation] Konkuk University Medical Center

[Name of Sponsor] Korea Health Industry Development Institute

Thank you for participating in the ‘Study on Patient Safety Education Needs’

This research is being conducted by the Ministry of Health and Welfare and the Korea Health Industry Development Institute (Research Title: Development of practical reflux method for improving patient safety level).

The purpose of this study is to identify and analyse the current state of patient safety education, educational needs and patient safety.

The survey will take about 20 minutes and there are no risks, losses, or benefits of medical conditions for those who are expected to participate in the survey. Also, under the guidance of the Research Ethics Committee, we do not collect your personal information (name, contact information, address). The survey will only be used for research purposes to develop patient and family educational content and will not be used for any other purpose.

You do not have to answer any questions that are uncomfortable, and you can stop participating in the research at any time during the investigation and there will be no disadvantages or discrimination.

-----------------------------------------------------

I have been fully informed of the survey. I have had the opportunity to ask questions about it and any questions that I have asked have been answered to my satisfaction. I consent voluntarily to participate as a participant in this research.

Print Name of Participant

Signature of Participant

Date

Day/month/year

**A. General Information**

*** Have you done any Patient Safety Officer work? ① Yes ② **No (☞** The survey ends.**)**

A1. At which hospital do you work?

① Tertiary hospital ② General hospital ③ Clinic ④ Long-term Care ⑤ Other ( )

A2. Does your hospital have a medical institution evaluation accreditation? (If applicable, check all)

① 1^st^ accreditation ② 2^nd^ accreditation ③ 3^rd^ accreditation ④ No accreditation

A3. How many beds does your hospital have?

① >200 beds ② 200 – 499 beds ③ 500 – 999 beds ④ ≥1000 beds

A4. What is your occupation?

① Nurse ② Doctor ③ Public health provider ④ General administrative staff ⑤ Other ( )

A5. Which department are you currently in?

① Department of Patient Safety and Quality Improvement ② Department of Infection Control ③ Ward ④ Other ( )

A6. Age:

A7. Gender ① Female ② Male

A8. Career duration in the hospital (years):

A9. Career duration as a Patient Safety Officer (years)

**B. State of patient safety education**

B1. Have you provided patient and family education? ① Yes ② **No (☞** Go to Question C.**)**

B1-1. If so, who did you educate?? (Multiple check)

1. Executive team ② Medical staff ③ Public health provider ④ General administrative staff ⑤ Patient and/or family ⑥ Other ( )

B2. What educational contents and methods did you provide?

| **Educational contents** | **Educational method** |
| --- | --- |
| 1) How to identify patients | ① Speak up ② Face-to-face ③ Campaign  ④ Templates, brochures, poster, etc.  ⑤ Video, YouTube, App, etc. ⑥ Other ( ) |
|  |  |
| 2) Handwashing methods | ① Speak up ② Face-to-face ③ Campaign  ④ Templates, brochures, poster, etc.  ⑤ Video, YouTube, App, etc. ⑥ Other ( ) |
|  |  |
| 3) How to prevent falls | ① Speak up ② Face-to-face ③ Campaign  ④ Templates, brochures, poster, etc.  ⑤ Video, YouTube, App, etc. ⑥ Other ( ) |
|  |  |
| 4) Hospitalisation orientation (hospital room etiquette, meal time, visit time, hospital facilities, fire prevention and evacuation tips, No smoking in hospital, etc.) | ① Speak up ② Face-to-face ③ Campaign  ④ Templates, brochures, poster, etc.  ⑤ Video, YouTube, App, etc. ⑥ Other ( ) |
|  |  |
| 5) Take all your medicines to your doctor visit (prescription medicines, vitamins, dietary or herbal supplements, etc.) | ① Speak up ② Face-to-face ③ Campaign  ④ Templates, brochures, poster, etc.  ⑤ Video, YouTube, App, etc. ⑥ Other ( ) |
|  |  |
| 6) Describe your medical condition (past history, allergy, surgery, etc.) | ① Speak up ② Face-to-face ③ Campaign  ④ Templates, brochures, poster, etc.  ⑤ Video, YouTube, App, etc. ⑥ Other ( ) |
|  |  |
| 7) Participate in your care plan | ① Speak up ② Face-to-face ③ Campaign  ④ Templates, brochures, poster, etc.  ⑤ Video, YouTube, App, etc. ⑥ Other ( ) |
|  |  |
| 8) Bring a list of questions to your doctor visits | ① Speak up ② Face-to-face ③ Campaign  ④ Templates, brochures, poster, etc.  ⑤ Video, YouTube, App, etc. ⑥ Other ( ) |

**C. Educational needs and patient safety**

C1. What is most needed to prevent medical errors?

① Patient and family engagement ② Employees’ patient safety activities ③ Patient safety culture ④ Budgeting of the hospital ⑤ Government support (National Health Insurance Fee for patient safety, National Patient Safety Campaign, etc.) ⑥ Other ( )

C2. Who do you think is most effective when educating patients and families?

① Patient and family engagement ② Patient Safety Officer ③ Medical staff ④ public health provider ⑤ general administrative staff ⑥ Other ( )

C3. What do you think is the most effective educational method?

① Speak up ② Face-to-face ③ Campaign ④ Templates, brochures, poster, etc. ⑤ Video, YouTube, App, etc. ⑥ Other ( )

C4. [**Providing health information**] What educational contents do you need for patients and families, and how much can it prevent medical errors?

| **Items** | **medical error prevention** | | | | | **Items** | **medical error prevention** | | | | |
| --- | --- | --- | --- | --- | --- | --- | --- | --- | --- | --- | --- |
| C4-1) Current medicines (including vitamins or dietary or herbal supplements) | **1** | **2** | **3** | **4** | **5** | C4-2) Allergies (drugs, food, pollen, dust, mites, etc.) | **1** | **2** | **3** | **4** | **5** |
|  | 1 (Never) -> 5 (Always) | | | | |  | 1 (Never) -> 5 (Always) | | | | |
| C4-3) Health history (medical history, past surgeries, etc.) | **1** | **2** | **3** | **4** | **5** | C4-4) Previous treatments/tests and complications associated with them | **1** | **2** | **3** | **4** | **5** |
|  | 1 (Never) -> 5 (Always) | | | | |  | 1 (Never) -> 5 (Always) | | | | |

C5. [**Speak up**] What educational contents do you need for patients and families, and how much can it prevent medical errors?

| **Items** | **medical error prevention** | | | | | **Items** | **medical error prevention** | | | | |
| --- | --- | --- | --- | --- | --- | --- | --- | --- | --- | --- | --- |
| C5-1) Handwashing (Ask if the staff washed their hands before performing a clean or aseptic procedure) | **1** | **2** | **3** | **4** | **5** | C5-2) Patient identification (Ask if patient identification was confirmed before medical practice) | **1** | **2** | **3** | **4** | **5** |
|  | 1 (Never) -> 5 (Always) | | | | |  | 1 (Never) -> 5 (Always) | | | | |
| C5-3) Asking about medical condition (‘What is my health problem?’ or ‘What is my diagnosis?’) | **1** | **2** | **3** | **4** | **5** | C5-4) Asking about test results (‘What is the test result?’ or ‘What are the possible complications?’) | **1** | **2** | **3** | **4** | **5** |
|  | 1 (Never) -> 5 (Always) | | | | |  | 1 (Never) -> 5 (Always) | | | | |
| C5-5) Asking about behaviour and changes in lifestyle (‘What should I eat?’ or ‘What exercises are good for me?’ or ‘What activities or foods should I avoid?’) | **1** | **2** | **3** | **4** | **5** | C5-6) Asking about the care plan (‘How many times perform the test?’ or ‘Why do I need this procedure?’ or ‘What is next my care plan?’) | **1** | **2** | **3** | **4** | **5** |
|  | 1 (Never) -> 5 (Always) | | | | |  | 1 (Never) -> 5 (Always) | | | | |
| C5-7) Asking about medicines (‘What will the medicine you are prescribing do?’ or ‘Are there reactions to this medicine?’ or ‘Can you change a medicine?’ or ‘How do I take it?’ or ‘Are there any side effects?’) | **1** | **2** | **3** | **4** | **5** | C5-8) Asking about medicine interaction (‘Will this medicine interact with medicines I’m already taking?’) | **1** | **2** | **3** | **4** | **5** |
|  | 1 (Never) -> 5 (Always) | | | | |  | 1 (Never) -> 5 (Always) | | | | |

C6. What do you think is a way to involve patients and families for patient safety? (Freely write)

|  |
| --- |
|  |
|  |

**Thank you for responding to our survey.**
